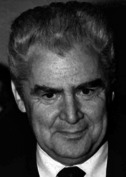# Permanganyl Fluoride: A Brief History of the Molecule MnO_3_F and of Those Who Cared For It

**DOI:** 10.1002/chem.202004759

**Published:** 2021-02-24

**Authors:** Hubert Schmidbaur, W. H. Eugen Schwarz

**Affiliations:** ^1^ Department Chemie Technische Universität München 85747 Garching Germany; ^2^ Department Chemie-Biologie Universität Siegen 57068 Siegen Germany; ^3^ Department of Chemistry Tsinghua University 100084 Beijing China

**Keywords:** permanganyl fluoride, permanganate, quantum chemistry, history of chemistry, extreme oxidation states

## Abstract

Permanganyl fluoride's existence at the stability threshold in the series of oxides and oxide fluorides of the late 3d transition metals is reflected by its experimentally challenging properties and by the difficulties posed in the theoretical description of its bonding characteristics. The history of this molecule is reviewed from early qualitative observations and the growing scattered information on its chemical and physical properties to the accurate determination and interpretation of its molecular structure and spectral features. The still problematic theoretical models for MnO_4_^−^ and MnO_3_F are briefly presented in the broader context of the chemistry of elements in high oxidation states. Short biographies of the scientists engaged in these studies are offered. Related technetium and rhenium compounds are briefly considered for comparison.

## Graphical abstract

**Permanganyl fluoride** is positioned at the stability threshold of the metal oxide fluorides of the 3d elements in their highest oxidation states. The history of this compound reflects the experimental and theoretical challenges faced by scientists over two centuries. The spectroscopic features of the molecule with its apparently simple closed‐shell configuration [Mn^7+^(d^0^), 3×O^2−^(p^6^), F^1−^(p^6^)] reveal an extremely complex electronic structure with non‐innocent ligands exerting an inverted ligand field.

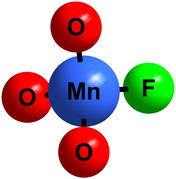

## Introduction

**Scheme 1:**
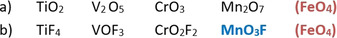
Metal oxides (a) and oxide fluorides (b) of elements Ti, V, Cr, Mn, Fe in their highest oxidation states. FeO_4_ is unstable under ambient conditions.

In a recent publication,^[1]^ the oxo chemistry of group VIII element iron in its highest oxidation states has been reviewed focusing in the end on the “non‐existence” of iron tetroxide FeO_4_, which could be the first and only example of a molecule containing iron it its highest oxidation state Fe^VIII^. Its absence from records of successful experiments is particularly intriguing since the tetroxides of the heavier congeners of iron are well documented as stable molecules RuO_4_ and OsO_4_. The absence of the cornerstone FeO_4_ is evident not only from the series of the transition metal oxides (Scheme 1 a), but also from that of the corresponding oxide fluorides (Scheme 1 b). A similar stability barrier like that between the iron tetroxide and the acid anhydride and acid halides of permanganic acid HMnO_4_ does not exist for the heavier elements of groups VII and VIII (Tc, Ru and Re, Os).

The two manganese(VII) compounds in the two series, Mn_2_O_7_ and MnO_3_F, were both first observed in the early 19th century. They could not be missed because both have deep colors, which quickly disappear in fierce explosions developing a dark brown smoke when samples are provoked by shock or heating. While the purple color of Mn_2_O_7_ differs only little from that of the permanganates M^I^[MnO_4_], the salts of the parent acid HMnO_4_, the short‐lived green color of MnO_3_F has been particularly intriguing to experimentalists, and later also to theoreticians. In contrast to the chemistry of Mn_2_O_7,_ which appears in all textbooks of introductory inorganic chemistry, information on permanganyl fluoride has remained more scattered. The present review therefore tries to summarize both the history and the present state‐of‐the‐art of this “weird molecule”. As in the text on the oxo chemistry of iron,^[1]^ the scientists engaged in the few studies carried out over a span of two centuries are also briefly introduced, because their biographies reflect the genealogical tree of brave chemists who had a special predilection for the challenge of working with this kind of compounds. Molecules such as MnO_3_F on the one hand fit well into the framework of simple models of structure and bonding, while on the other hand they pose a challenge both for the experimentalist and the theoretical chemist. In a final chapter therefore the status of the theoretical description of the electronic structure is up‐dated here.

To complement the story, and to place the information in a broader context, the literature on the corresponding chloride, MnO_3_Cl, and on the technetium analogue, TcO_3_F, have also been included in a concise format. The chemistry of rhenium(VII) compounds is not considered because it follows more conventional rules and is devoid of similar irregularities.

## A Brief History of Permanganyl Fluoride, MnO_3_F

**Figure 1:**
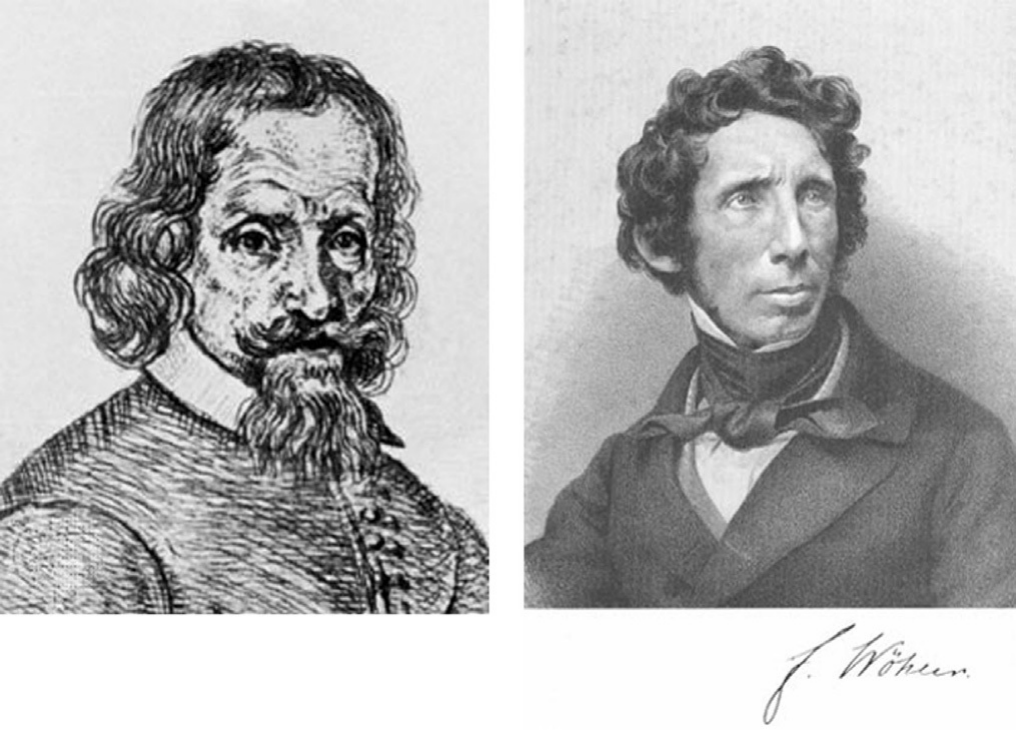
*Johann Rudolph Glauber* (left, 1604–1670) lived, studied, and worked as a pharmacist and chemical engineer at various places in the German speaking European countries. He first observed the deeply colored permanganates in 1659, when he reacted the mineral pyrolysite (MnO_2_) with sodium carbonate in air. He obtained a melt containing the green sodium manganate Na_2_MnO_4_, which was converted upon neutralization in water into sodium permanganate NaMnO_4_ with a color he described as “elegans color purpureus”. Crystallization was optimized with the addition of pottash to give KMnO_4_.^[6]^ Its crystal structure has been determined by Gus Palenik (University of Waterloo) in all its details as late as 1967. In the crystal, the slightly distorted tetrahedral anion has Mn−O bonds of 1.607(5) Å.^[7]^ No further biographic details of Johann F. Glauber and of Friedrich Wöhler (right, 1800–1882, a founder of the modern chemical sciences) are described here because their brief encounters with the subject are only small anecdotes in their immense contributions to science.

**Figure 2:**
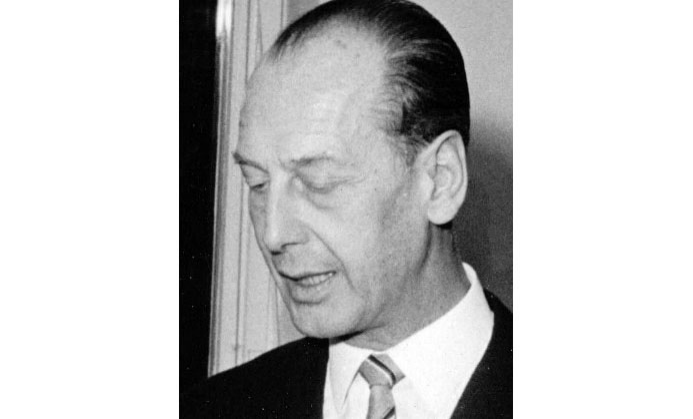
*Oskar Glemser* (1911–2005) studied Chemistry at the Technische Hochschule Stuttgart and held positions at the RWTH Aachen before he took the chair of inorganic chemistry at Göttingen where he dedicated much of his work to fluorine chemistry, attracting in particular several young talents to this field (see below). As the president of the Göttingen Academy of Sciences and of the German Chemical Society, in his time he was one of the most prominent figures in chemistry.

The acid halides of permanganic acid have remained what is widely called laboratory curiosities for almost two centuries. According to two reports published in 1828, it was Friedrich Wöhler (Figure 1, right) at the University of Göttingen who first observed a deep green color which appeared upon treatment of purple potassium permanganate with strong acid containing fluoride, that is, by reacting a mixture of KMnO_4_ and CaF_2_ with sulfuric acid [Eq. 1]. The acid took on a green color, and a green vapor was evolved which exploded to give a deep‐brown smoke.[^2^, ^3^] After these early observations Wöhler never revisited this compound. It had previously long been known that potassium permanganate KMnO_4_ when treated with sulfuric acid affords the highly explosive anhydride Mn_2_O_7_ of permanganic acid [Eq. 2].^[4]^ Therefore the instability of the acid fluoride was not unexpected. The first more extensive description of the properties of Mn_2_O_7_ was provided by Oskar Glemser of the University of Göttingen in 1953 (Figure 2).^[5]^

(1) $$2\;\mathrm{KMnO}{}_{4}+2\;H{}_{2}\mathrm{SO}{}_{4}+\mathrm{CaF}{}_{2}\to \;\;\;\;\;\;\;2\;\mathrm{MnO}{}_{3}F+K{}_{2}\mathrm{SO}{}_{4}+\mathrm{CaSO}{}_{4}+2\;H{}_{2}O$$

(2) $$2\;\mathrm{KMnO}{}_{4}+H{}_{2}\mathrm{SO}{}_{4}\to \mathrm{Mn}{}_{2}O{}_{7}+K{}_{2}\mathrm{SO}{}_{4}+H{}_{2}O$$

**Figure 3:**
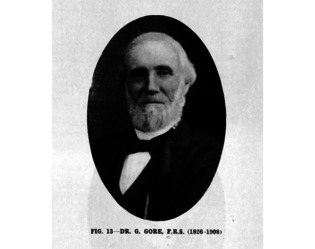
*George Gore* (1826–1908) was born as the son of a workman in Bristol and as a young boy was an apprentice to become a copper‐ and blacksmith. Fascinated by the wonders of science, he started educating himself and soon was drawn to the city of Birmingham with her rapidly growing chemical industries. There he first joined a phosphorus factory, and the invention of the “safety match” has been attributed to him for the work he carried out there. This and other discoveries in typefounding, dyeing, electroplating and other trades made him a fortune. He was later appointed director of an institute of scientific research in Birmingham where he guided many programs of chemical technology. Except for a pension for his daughter, he dedicated his rich belongings to the Royal Society and the Royal Institution.

Half a century after Wöhler's observations, George Gore FRS (Figure 3), working at Birmingham, reported that “damp crystals of permanganate of potassium hissed and dissolved to a green solution” when reacted with hydrofluoric acid [Eq. 3].^[8]^ This experiment was just one of the studies he carried out with hydrofluoric acid, a product which he commercialized. His biography shows that this observation was by no means Gore's greatest contribution to science and technology.

(3) $$\mathrm{KMnO}{}_{4}+3\;\mathrm{HF}\to \mathrm{MnO}{}_{3}F+\mathrm{KF}\cdot \mathrm{HF}+H{}_{2}O$$

**Figure 4:**
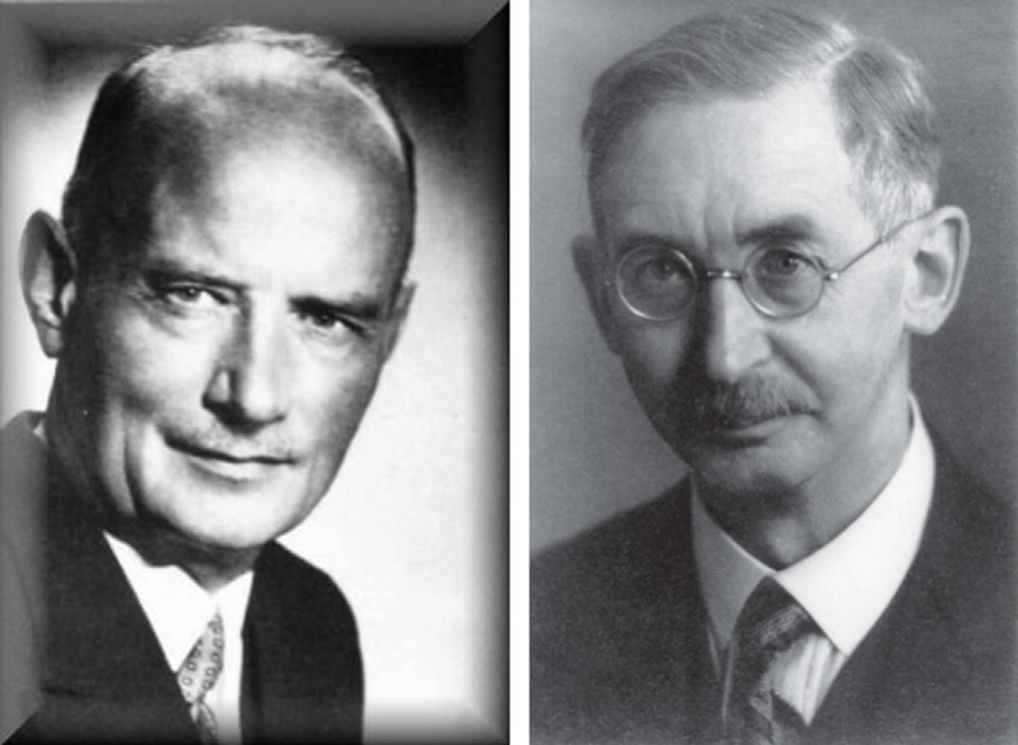
*Otto Ruff* (left, 1871–1939) in his time was one of the protagonists of fluorine chemistry in Europe. He had started as a pharmacist, and obtained his PhD with Oskar Piloty in Berlin, but then first joined Emil Fischer who had newly arrived in Berlin at the turn of the century. After a brief period with Wilhelm Ostwald in Leipzig, where he received important training in physical chemistry, he returned to Berlin before he was awarded his first chair of inorganic chemistry at Danzig (now Poland). His work on fluoro‐ and chlorosulfonic acids was only a minor activity at a time when he also worked not only on the synthesis of diamonds, but also isolated uranium hexafluoride for the first time (1909). After 10 years he took a chair at the University of Breslau (now Poland), where he retired. Ruff's carefully documented data on UF_6_ founded the basis for much of the later work carried out under the Manhattan Project in the early 1940s in the US, where Aristid von Grosse was one of the prominent scientists (see below). *Karl Fredenhagen* (right, 1877–1949) was born in Loitz (near Greifswald) in 1877, and had studied chemistry at the University of Göttingen and received his PhD with Walther Nernst. He later worked also with Wilhelm Ostwald at the University of Leipzig. His most widely known work on hydrogen fluoride led to a new process for the production of elemental fluorine documented in a valuable patent in 1928. In yet another area of his broad interests, he was one of the pioneers in the development of potassium graphite KC_8_ which up to the present is an extremely important material in the battery industry and related technologies.

It then took another half century until Otto Ruff (Figure 4, left), then working at the University of Danzig, in his first studies of fluorosulfonic acid included the reaction with permanganate.^[9]^ He again observed that the evolved vapors, which sometimes were violet, exploded vehemently upon shock and slight warming. It led him to assume that the volatile product was only manganese heptoxide and would probably not contain any fluorine.

While Otto Ruff thus missed out in his search for MnO_3_F, Karl Fredenhagen (Figure 4, right), working at the University of Greifswald, 20 years later confirmed the formation of green vapors from the reaction of KMnO_4_ with hydrofluoric or fluorosulfonic acid that may be formulated by Equations (3) and 4a/4b).^[10]^ He assigned the color to a manganese oxyfluoride with manganese in the oxidation state +VII, but gave no formula. It should be noted that a second publication (of 1939) has Helmut Fredenhagen as the author, who also worked at Greifswald and who thanked Karl Fredenhagen for the guidance.^[11]^

(4a) $$\mathrm{KMnO}{}_{4}+\mathrm{HSO}{}_{3}F\to \mathrm{MnO}{}_{3}F+\mathrm{KHSO}{}_{4}$$

(4b) $$\mathrm{KMnO}{}_{4}+2\;\mathrm{HSO}{}_{3}F\to \mathrm{MnO}{}_{3}F+\mathrm{KSO}{}_{3}F+H{}_{2}\mathrm{SO}{}_{4}$$

Relevant work carried out during and after World War II in the Greifswald laboratories was published in 1950 by Kurt Wiechert (Karl Fredenhagen had died in 1949).^[12]^ As described therein, a second method was employed for the generation of MnO_3_F starting this time from manganese metal which was treated with potassium nitrate in hydrogen fluoride. Both green vapors and green solutions were again observed. Fractional condensation of the volatiles at −78 °C afforded a deep‐green, almost black solid which melted upon warming to −45 ° to a green liquid and decomposed violently near 0 °C. The elemental analysis of the condensate gave an atomic ratio of Mn:F = 1:1 with percentages approaching those calculated for MnO_3_F confirming finally all previous suggestions. However, reconsidering the properties given for the compound at this stage, the products still were not really pure MnO_3_F.

In 1950, Aynsleigh, Peacock and Robinson of the University of Newcastle‐upon‐Tyne studied the reaction of elemental fluorine, diluted with nitrogen, with manganese oxides and potassium permanganate at 150 °C and found no evidence for a volatile MnO_3_F.^[13]^ This result led them to question the earlier evidence by Wöhler and all the others mentioned above. This failure no doubt was due to the applied elevated temperature well beyond the stability range of MnO_3_F.

**Figure 5:**
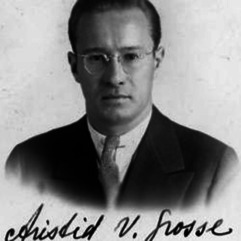
The family of *Aristid v. Grosse* (1905–1985) was of Baltic origin, his father being a diplomat of the Tsar government assigned to the Russian embassy in Shanghai. He was born 1905 in Riga (Latvia then being part of the Tsar's empire), but received all his education in China and Japan before he enrolled at the Technische Hochschule in Berlin and received his PhD in the chemical sciences. He then held a post‐doctorate position at the Kaiser Wilhelm Institute in Berlin working with Otto Hahn and succeeded in the first preparation of protactinium metal. The various isotopes of element 91 had previously been detected by Kasimir Fajans, Otto H. Göhring, Otto Hahn, and Lise Meitner. He emigrated to the United States in 1930 and worked at the University of Chicago and at Columbia University, New York, where in the 1940s he became engaged in the Manhattan Project dealing mainly with the chemistry of the uranium fluorides. As mentioned above, in the chemistry of uranium hexafluoride he stood on the shoulders of Otto Ruff. For his achievements in fluorine chemistry of the actinides he received a United States Atomic Energy Commission Award in recognition of his “outstanding contributions to the development of nuclear energy.” In 1948 he took on the position of the director of the Research Institute of Temple University and of the Franklin Institute in Philadelphia, where he retired.

Only a few years later (1954), Alfred Engelbrecht and Aristid von Grosse (Figure 5), working at Temple University in Philadelphia,^[14]^ were finally able to isolate MnO_3_F in a pure state and to determine its fundamental properties. The compound was obtained from potassium permanganate and fluorosulfonic acid in a molar ratio of 1:4 in a copper flask cooled to −78 °C, and separated in a vacuum by fractional condensation [Eq. (4b) with an excess of HSO_3_F]. In the reaction, HF is formed in small quantities presumably through the reaction formulated in Equation 5.

(5) $$H{}_{2}\mathrm{SO}{}_{4}+\mathrm{HSO}{}_{3}F\to H{}_{2}S{}_{2}O{}_{7}+\mathrm{HF}$$

These traces of HF remaining in the green condensate were removed by the addition of KF which absorbs HF to form KHF_2_ but is inert towards MnO_3_F. The green crystals melt at −38 °C and the liquid has a vapor pressure of 22.5 mm at −15 °C and of 52 mm at 0 °C. The extrapolated boiling point is +60 °C. When kept above 0 °C, the compound decomposes slowly, or occasionally violently, with formation of a flame and a brown smoke. In moist air, violet vapors of Mn_2_O_7_ appear prior to violent explosions. With excess water more carefully applied, HMnO_4_ and HF are formed [Eq. 6]. This hydrolysis was used for the elemental analysis of the compound, which gave the expected values for Mn and F. It is remarkable that the authors dared to prepare up to 40 g quantities (!) of this highly explosive material.

(6) $$\mathrm{MnO}{}_{3}F+H{}_{2}O\to \mathrm{HMnO}{}_{4}+\mathrm{HF}$$

In later work, both von Grosse and Engelbrecht, in collaboration with A. Javan of Columbia University in New York, studied the microwave spectrum of MnO_3_F, and the molecular constants of the symmetric spinning top molecule (point group *C*_3*v*_) have been determined.[^15^, ^16^, ^17^] In the gas phase the molecule shows three symmetry‐related Mn−O distances of 1.586 Å and an Mn−F distance of 1.724 Å, with O‐Mn‐F angles of 108.27°. The quadrupole hyperfine structure confirmed the nuclear spin of 5/2 for the only stable ^55^Mn isotope (100 % abundance) with a quadrupole coupling constant of eq*Q* = 16.8 Mc sec^−1^ and a nuclear quadrupole moment of *Q* = 0.55×10^−24^ cm^2^. Similar studies were carried out for perrhenylfluoride ReO_3_F.[^18^, ^19^] In more recent work, the electrical field gradients of the metal atom in MnO_3_F were calculated using density functional theory by Bjornsson and Bühl.^[20]^

The question arises why this highly decorated protagonist of Nuclear Chemistry would care for the synthesis and properties of permanganyl fluoride in 1953. An answer to this question becomes apparent from the social contacts during his days at the Kaiser‐Wilhelm (now Max Planck) Institute in Berlin. It was there that he also met Ida Tacke and Walter Noddack who had discovered (together with O. Berg) the element rhenium in 1925, one of the two elements that Mendelejev had predicted half a century ago as the yet to be discovered congeners of manganese. Eka‐manganese was still missing and was discovered only in 1937 and named technetium. Manganese was the prototype of the intriguing triad Mn‐Tc‐Re, and when finally all three elements had become available it was of natural interest to a senior scientist in the field to study all the fundamentals of their chemistry. It is fair to say that all later work on the elusive MnO_3_F molecule was based on the experimental progress made by A. v. Grosse and A. Engelbrecht. These two authors also first produced pure chromyl fluoride CrO_2_F_2_, which is even more volatile than the “isoelectronic” and “isosteric” permanganyl fluoride, but also much more stable being nonexplosive.^[21]^ The orange crystals of CrO_2_F_2_ melt at 31.6 °C and the vapor pressure reaches 760 mm Hg already at 29.6 °C. The gas phase structure has been determined.[^22^, ^23^, ^24^]

Alfred Engelbrecht was born in 1923 in a small village in Tyrolia and was educated for his matura in Innsbruck, Austria. Being drafted in the early days of World War II, he was severely injured and allowed to return to Innsbruck in 1944 to study chemistry there for his PhD (1948). Thereafter he went to the US on one of the early UNESCO programs “to gain expertise in modern fluorine chemistry” and had post‐doctorate positions with George H. Cady at the University of Washington in Seattle and with Aristid von Grosse in Philadelphia. He returned to Innsbruck in the early 1950s for his habilitation for which—small wonder—he chose to work on perchloryl fluoride ClO_3_F on which he first published in 1952.^[25]^ Colorless perchloryl fluoride (m.p. −152.2 °C, b.p. −48.1 °C) has a sweetish odor, but is highly toxic. It is thermally much more stable than permanganyl fluoride and decomposition starts as high as 450 °C. The compound was long considered as a component for powerful rocket propellants. In 1965 Engelbrecht was offered a chair at the University of Innsbruck and established a highly productive school of fluorine chemists there. He had to take early retirement owing to the handicaps inflicted by his war injuries. Unfortunately, we did not find a photograph of Albert Engelbrecht on public records.

**Figure 6:**
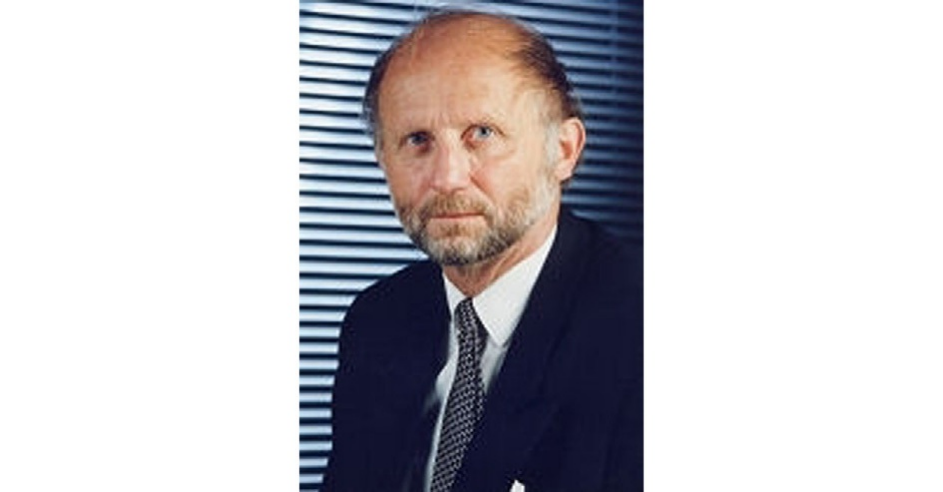
*Achim Müller* (*1938) studied chemistry at the University of Göttingen and received his PhD with Oskar Glemser, where he became acquainted with fluorine chemistry. Later he contributed pioneering work to the spectroscopy of small inorganic molecules and ions focusing mainly on oxo complexes of the transition metals in their high oxidation states. His later work on polyoxometallates is legendary.

Starting in 1969, the group of Achim Müller (Figure 6) at the University of Dortmund and later at Bielefeld studied for the first time both the electronic absorption spectrum[^26^, ^27^] and the IR spectrum of MnO_3_F in the gas phase,[^28^, ^29^] followed by work with the emerging technique of He^I^ photoelectron spectroscopy.^[30]^ Data from all these analytical techniques had still been missing. The results explained the deep green color of the compound arising from O → Mn charge transfer absorptions similar to the corresponding transitions of the purple permanganate ion with its idealized *T_d_* symmetry, which is lowered to point group *C*_3*V*_ for MnO_3_F (see below). In all their experiments, the Müller group used the von Grosse–Engelbrecht method for the preparation of their samples.

M. J. Reisfeld, L. B. Asprey, and N. A. Matviyoff (University of California, Los Alamos Scientific Laboratory) also studied the IR spectrum of gaseous MnO_3_F and (in 1971) carried out a more detailed vibrational analysis determining the valence force constants. The fundamental wave‐numbers (in cm^−1^) of MnO_3_F are: *A*_1_ = 905.2 (dominantly: symmetric M−O stretch), 720.7 (Mn−F stretch), 337.7, *E =* 952.5 (anti‐symmetric M−O stretch), 373.9 and 264.3 cm^−1^, with Coriolis coupling constants of ζ_4_ = 0.10, ζ_5_ = −0.03, ζ_6_ = 0.37.^[31]^ In 1975, this work was followed by another study carried out by J. P. Jasinski, S. L. Holt, J. H. Wood, and J. W. Moskowitz at the University of Wyoming on the calculated and experimentally determined electronic structure of gaseous MnO_3_F.^[32]^ This study was further complemented in 1976 by another microwave investigation of MnO_3_F published by J. Høg and T. Pedersen of the University of Copenhagen.^[33]^ In 1991, A. L. Brisdon, J. H. Holloway, E. G. Hope, P. J. Townson, W. Levason, and J. S. Ogden at the Universities of Leicester and Southampton presented a more comprehensive study of the UV/Vis spectra of manganese and rhenium oxide fluorides in low‐temperature matrices.^[34]^ The resonance Raman spectrum of MnO_3_F has been registered by E. L. Varetti at liquid air temperature using a laser scanning technique, and the harmonic wavenumbers and anharmonicity constants have been determined.^[35]^

**Figure 7:**
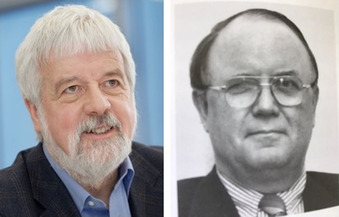
*Walter Thiel* (left, 1949–2019) studied chemistry at the University of Marburg and spent sabbatical years with Michael J. S. Dewar at the University of Texas at Austin. After positions in Wuppertal and Zürich he joined the Max Planck Institute in Mülheim where he was actively involved in a diversity of projects. He died untimely in 2019. *Hans Bürger* (right, *1937) studied chemistry at the RWTH Aachen and received his PhD with Ulrich Wannagat. During a stay with Hermann J. Becher at the Technical University of Stuttgart he became interested in the vibrational spectroscopy of inorganic molecules (in the tradition of Becher's mentor Joseph Goubeau, both of which had their start at Göttingen). Hans Bürger continued this work first at the Technical University of Braunschweig and finally in Wuppertal where he retired. Throughout his later career he suffered from the consequences of a fierce explosion in the laboratory affecting mainly his eyes.

The most recent experimental study of the high resolution vibrational spectra of MnO_3_F in both the ground and first excited state, followed up by gradient‐corrected DFT calculations, has been carried out by the groups of Walter Thiel and Hans Bürger (Figure 7) at the University of Wuppertal and the University of Zürich, respectively. A complete set of molecular parameters and vibration‐rotation coupling constants has been presented.^[36]^ The molecular structure determined by the microwave analysis^[15]^ has been well reproduced by single‐configuration density‐functional corrected BLYP and BP86 calculations, while ab initio configuration‐mixing methods still turned out to be less satisfactory. MnO_3_F was the first polyatomic molecule where this had been demonstrated not only for the harmonic wave numbers, but also for the anharmonic spectroscopic constants.

**Figure 8:**
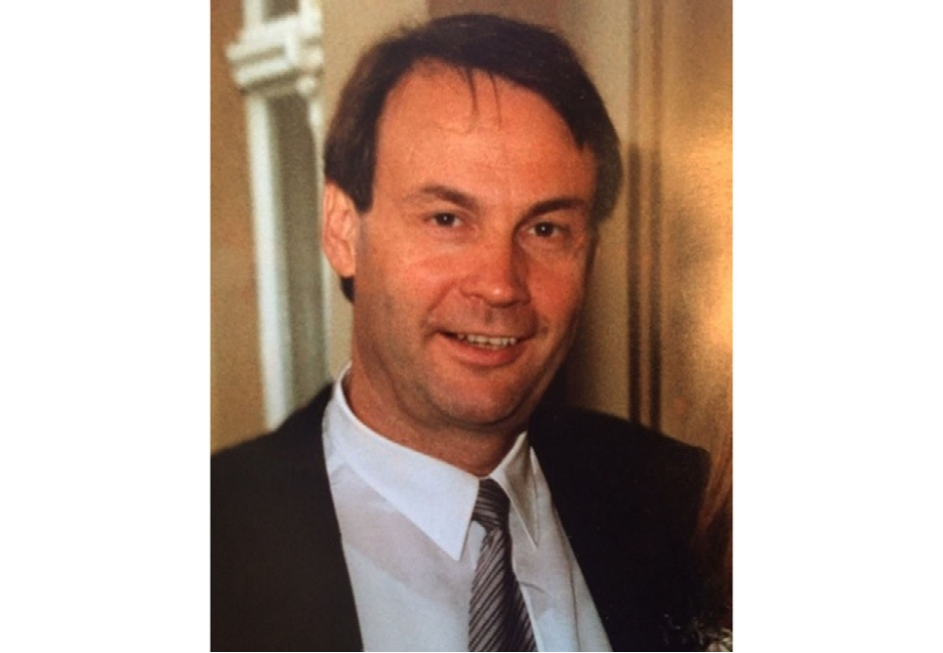
*Konrad Seppelt* (*1944) received his doctorate degree at the University of Heidelberg with Wolfgang Sundermeyer, another former student of Oskar Glemser, followed by a post‐doctorate position with Neil Bartlett at UC Berkeley where he became even more fascinated by fluorine chemistry. During his more than 30 years at the Free University of Berlin (1980–2010) he made many highly important contributions to the chemistry of elements in their highest oxidation states and has been a protagonist of this area of chemistry.

In 2006, the group of Konrad Seppelt (Figure 8) at the Free University of Berlin has finally determined the crystal structure of MnO_3_F at −120 °C.^[37]^ Single crystals were grown from liquid HF at −78 °C (monoclinic, space group *C*2/*c*, *Z* = 4). Owing to the small differences in the Mn−O and Mn−F distances, and because the O and F atoms have a similar (negative) charge according to a Mulliken analysis, the molecule is heavily disordered in the crystal and no specific sites can be distinguished for the O and F atoms. The average Mn−O/F distance was found to be 1.621 Å, in good agreement with the gas phase microwave data (3× 1.586 and 1× 1.724 Å).^[15]^ In a K‐edge EXAFS (extended X‐ray absorption fine structure) study carried out by groups at the universities of Southampton and Leicester the M−O and Mn−F bond lengths were found to be 1.59 and 1.72 Å.^[38]^ A molecular orbital description of the core excitation spectrum was provided by ab initio CI calculations published by Decleva, Fronzoni, Lisini, and Stener of the University of Trieste.^[39]^

## A Glimpse on Related Molecules

### Permanganyl chloride MnO_3_Cl

Permanganyl chloride was first mentioned in a report by B. Franke (University of Leipzig) on oxides and halides of manganese in 1887^[40]^ indicating a possible relation to Wöhler's fluoride, but without a clear description. No less than a century later, D. Michel and A. Doiwa reacted potassium permanganate in sulfuric acid with gaseous HCl and obtained a green‐violet volatile product that was characterized by its UV/Vis spectrum.^[41]^ This study was followed by an investigation of T. S. Briggs at Cornell University who reacted potassium permanganate with chlorosulfuric acid at −60 °C [Eq. 7].^[42]^ MnO_3_Cl is a volatile liquid, which solidifies near −68 °C. The color of solutions in CCl_4_ is pinkish orange, but the pure liquid is almost black and deflagrates explosively when allowed to warm above 0 °C, while slow hydrolysis yields HCl and HMnO_4_.

(7) $$\mathrm{KMnO}{}_{4}+\mathrm{HSO}{}_{3}\mathrm{Cl}\to \mathrm{MnO}{}_{3}\mathrm{Cl}+\mathrm{KHSO}{}_{4}$$

Soon thereafter the group of Achim Müller studied the UV/Vis absorption spectrum of MnO_3_Cl,^[26]^ followed by the study of its He^I^ photoelectron spectra.^[30]^ In a most thorough study carried out by the Seppelt group at the Free University of Berlin^[37]^ the compound was prepared by the reaction of HSO_3_Cl and KMnO_4_ at −30 °C, separated by vacuum fractionation and finally crystallized at −196 °C. Its crystal and molecular structure have been determined by single crystal X‐ray diffraction (orthorhombic, space group *Cmc*2_1_, *Z* = 4). The crystals are green at −150 °C, while the liquid is dark brown at −100 °C. This color change is probably due to the ordered organization of the molecules in the crystal, where short Cl–Cl contacts are established which affect the charge transfer excitations. The average bond lengths are 1.586 Å for Mn−O and 2.099 Å for Mn−Cl. This much larger difference induces the ordering of the molecules in the crystal, whereas for MnO_3_F disorder had been observed. A Mulliken charge analysis suggests that the bonds are inversely polarized as (O_3_Mn)^+δ^−F^−δ^ and (O_3_Mn)^−δ^−Cl^+δ^, with *δ* ≈ 0.2, reflecting the larger electronegativity of F as compared to Cl, and the large electronegativity of Mn^VII^ attached to three O atoms. The positive charge at the Cl atoms and their charge deformation makes them electro‐ and nucleophilic, which is borne out by the intermolecular Cl–Cl donor/acceptor contacts in the crystals. The Raman spectrum has been observed for the liquid at −120 °C, and the six strong bands at 948 (dominantly: anti‐symmetric Mn−O stretch), 887 (symmetric Mn−O stretch), 457 (Mn−Cl stretch), 365, 306, and 217 cm^−1^ have been assigned to the vibrations of the molecule with *C*_3*v*_ symmetry, supported by DFT calculations.^[37]^

### Pertechnetyl fluoride TcO_3_F

The history of TcO_3_F is much shorter. The compound was first synthesized in weighable amounts by H. Selig and G. Malm in 1963 at Argonne National Laboratory by the reaction of TcO_2_ with elemental fluorine and described as a volatile yellow solid melting at 18.3 °C. The extrapolated boiling point is 100 °C.^[43]^ In a subsequent paper, the Selig group, working at the Hebrew University in Jerusalem, described the synthesis from ammonium pertechnetate and hydrogen fluoride [Eq. 8] and reported IR and Raman data of the compound in the gas and liquid phase, respectively. The bands were assigned to the monomeric molecule with *C*_3*v*_ symmetry. The ^19^F resonance has also been recorded at δ +50 ppm (vs. CCl_3_F). No coupling with the ^99^Tc isotope (*I* = 9/2) was observed^[44]^ [Eq. 9]:

(8) $$\mathrm{KTcO}{}_{4}+3\;\mathrm{HF}\to \mathrm{TcO}{}_{3}F+\mathrm{KF}\cdot \mathrm{HF}+H{}_{2}O$$

(9) $$\mathrm{KTcO}{}_{4}+4\;\mathrm{HF}+\mathrm{BiF}{}_{5}\to \mathrm{TcO}{}_{3}F+\mathrm{KF}\cdot \mathrm{HF}+H{}_{3}O{}^{+}\;\mathrm{BiF}{}_{6}{}^{-}$$

**Figure 9:**
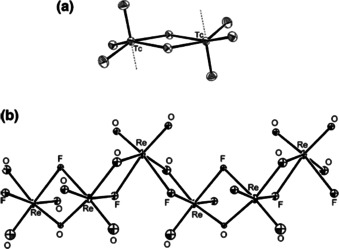
Molecular structures in the solid state. (a) [TcO_3_F]_2_ (the vertical dotted lines mark connections to O‐atoms of neighboring dimers). (b) [ReO_3_F]_*n*_.

The group of Seppelt also studied the Raman spectrum of TcO_3_F and determined the solid state structure by single crystal X‐ray diffraction at −100 °C. The compound has been shown to be weakly associated into a dimer with fluoride bridging between the monomers. Further weak interactions with oxygen atoms of neighboring dimers lead to an extended array. The structure is therefore intermediate between MnO_3_F, which is strictly monomeric, and ReO_3_F, which is a polymer in the crystal (Figure 9). The assignment of the vibrational spectra has been revised accordingly. The samples for this study were obtained by treating KTcO_4_ with HF in the presence of excess BiF_5_ added to capture the water produced in the process as H_3_O^+^ BiF_6_^−^ [Eq. (9)]. In this work, the crystal structures of CrO_2_F_2_ and VOF_3_ have also been determined.^[45]^

### Pertechnetyl chloride TcO_3_Cl

The preparation of pertechnetyl chloride TcO_3_Cl (in several papers also named pertechnyl chloride) has been achieved via several routes including the reaction of technetium chlorides with oxygen and of technetium metal with chlorine and a non‐specified origin for oxygen. The product was described as a volatile yellow liquid.[^46^, ^47^] In several reports the vibrational and the vapor phase UV/Vis absorption spectra have been described and assigned in comparison with those of the rhenium analogue. The model of mononuclear molecules with *C*_3*v*_ symmetry allowed a consistent analysis of the experimental data.[^48^, ^49^, ^50^] Later work was also dedicated to the mass spectral behavior of MnO_3_Cl and related compounds and their adsorption properties on surfaces.^[51]^ The electronic structure of the gas phase monomer has been calculated by relativistic DFT methods,^[52]^ but the crystal structure has not yet been determined.

### The oxides Mn_2_O_7_, Tc_2_O_7_ and Re_2_O_7_

**Figure 10:**
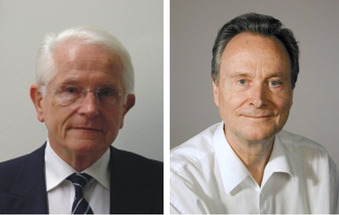
*Bernt Krebs* (*1938) started his career at the University of Göttingen, where he worked with Gerhard Gattow. He broadened his expertise during a post‐doctorate stay at Brookhaven National Laboratory. After appointments at the Universities of Kiel and Bielefeld, he established a large school at the University of Münster where he worked on a broad spectrum of research areas including also mainly oxometallates of the late transition elements. Arndt Simon (*1940) studied chemistry at the University of Münster and received his PhD in 1966 with Harald Schäfer. From a faculty position there, he was appointed director of the Max Planck Institute of solid state research in Stuttgart and became affiliated with the University of Stuttgart. His work has spanned many disciplines of preparative and structural inorganic chemistry, and his encounter with Mn_2_O_7_ is just one example, how he was always attracted by the most delicate research problems.

As mentioned above,[^4^, ^5^] Mn_2_O_7_ has a long history. However, its crystal and molecular structure have been determined as late as 1987 by a joint work of the groups of Arndt Simon and Bernt Krebs (Figure 10) working at the MPI in Stuttgart and at the University of Münster,^[53]^ respectively, where the crystal structure of Tc_2_O_7_ had first been elucidated earlier by the Krebs group.^[54]^ The interesting structural properties of this compound have recently been revisited.[^55^, ^56^] The structure of Re_2_O_7_ had also been known from early work by Bernt Krebs.^[57]^ In many details, the structural results of the dinuclear units (for Mn, Tc) and the coordination polymer (for Re) of the M_2_O_7_ heptoxides resemble those for the corresponding acid fluorides MO_3_F.

## Current View of the Electronic Structure of Permanganate and Permanganyl Fluoride

The multifaceted history of permanganyl fluoride would be incomplete without a short account of the current view of its electronic structure. The MnO_3_F molecule is an example that throws light both on the helpful role of the molecular orbital and ligand field models to better understand the bonding situation in a molecule, and on their limitations.

As an introduction to the subject it is worth to quote a *caveat* from a paper by Tom Ziegler published in 2012 where he writes: “The notion of permanganate being an easy system is completely wrong.“^[58]^ If this is true, permanganyl fluoride will also not be an easy task. This situation may become evident from the following summary of pertinent aspects.

In chemistry's “standard model” of molecular electronic structure, it is assumed that the atomic and molecular valence shells can be approximated as a set of N single electrons in N, more or less localized, spin‐orbitals (or N/2 spin‐coupled pairs in N/2 orbitals). In the Born–Oppenheimer approximation, the dynamical molecular structure is more or less accurately represented by a fixed geometrical arrangement of the nuclei. The quantum‐chemical electronic wavefunction is approximated by a single N‐electronic Slater determinant of N spin‐orbitals which can often be depicted or cartooned by a chemical Lewis formula. However, it may happen that the observed structural, spectroscopic and reactivity parameters cannot be consistently combined into such a model. From the experimental side, a more general model would be preferable in such cases. From the theoretical side, a reliable analysis of the model defects is desired.

**Figure 11:**
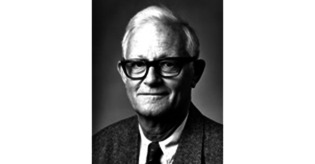
*Carl Johan Ballhausen* (1926–2010) was born in København in 1926 and studied chemistry there with Niels J. Bjerrum. After a serious traffic accident in 1949 and during the long hospitalization, he made the best of this situation and taught himself quantum mechanics for use in chemistry. After assistantships at Harvard, the Bell Telephone Laboratories, and the University of Chicago, he eventually became professor (from 1959–1996) and director of the Institute of Physical Chemistry of the University of Copenhagen. Ballhausen contributed deeply to the renaissance of inorganic chemistry after World War II, merging the fields of spectroscopy, structural chemistry, and molecular quantum mechanics. He combined the symmetry aspects of the electrostatic “crystal field theory” with the physical meaning of the parameters of “ligand field theory”, incorporating metal–ligand orbital overlap and introducing molecular orbital models of covalent bonding.

An important step toward the understanding and interpretation of electric, magnetic and optical properties of transition metal compounds was undertaken around 1930 by Becquerel,^[59]^ Bethe^[60]^ and van Vleck.[^61^, ^62^] In their crystal field theory, it were basically the symmetry aspects of the systems that were exploited. In the 1950s, this line was resumed by Ilse and Hartmann,[^63^, ^64^] Griffith and Orgel^[65]^ and merged with Mulliken's^[66]^ concept of “perturbation” by the mixing of atomic orbitals to form molecular orbitals, following the early suggestion of Van Vleck[^67^, ^68^] of 1935. This advance was summarized in the famous book of Ballhausen^[69]^ in 1962 (Figure 11; compare also Schläfer und Gliemann, 1967).^[70]^

**Scheme 2:**
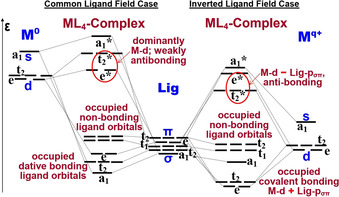
Schematic orbital level diagram for transition metal complexes of the type ML_4_ with a tetrahedral structure. Left: Traditional order for “M^0^” in zero or low oxidation state, with the occupied ligand (“Lig”) orbital levels energetically below the partially occupied M‐d orbitals. Right: Alternative order for “M^*q*+^” in high oxidation state, with ligand and (nearly) empty M‐d orbitals.

The crystal field and adapted ligand field models worked well for complexes of transition metals (M) *in their lower and medium oxidation states*. As an example, the common one‐electron orbital level Scheme for complexes of type ML_4_ with a tetrahedral structure is shown on the left‐hand side of Scheme 2. The occupied valence levels are of dominant ligand L‐2p type (in the case of oxides and fluorides, L = O or F). The lowest orbitals are stabilized by admixtures of M‐d and M‐s atomic orbitals. The scheme represents the dative bonding interaction by the four L‐2p(σ) pairs, donated (in the case of the first row of the transition metals) into the partially occupied M‐3d(t_2_) and unoccupied M‐4s(a_1_) parts of the valence‐shell of the central metal atom, enhanced by weaker L‐2p(π) donation into M‐3d(e). In tetrahedral complexes, the d(e) orbitals point toward the ligands, and the d(t_2_) orbitals are directed between the ligands. This yields the energy order of orbital symmetries a_1_ < t_2_ < e, according to Ballhausen's ligand field concept.[^69^, ^70^, ^72^] The upper set of six partially occupied or unoccupied valence orbitals forms the mirror image e* < t_2_* < a_1_*, predominantly consisting of M‐3d and M‐4s atomic orbitals. The intermediate range of energy levels holds six occupied L‐2p ligand orbitals, from where UV‐ionizations or excitations may occur (L → M charge transfer).

**Figure 12:**
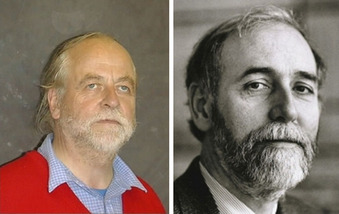
*Tom Ziegler* (left, 1945–2015) studied theoretical chemistry with C. J. Ballhausen at the University of Copenhagen where he received a PhD degree in 1972. Most of his life he worked in Canada, where he acted as a professor of theoretical chemistry at the University of Calgary since 1986. In recent years he had regularly stayed as a guest researcher at the Mulliken Center in Bonn, where he unexpectedly died in 2015. He is widely known for his contributions to the development of density functional theory and the coding of the Amsterdam Density Functional software developed in the group of E. J. Baerends, and for applying it to transition metal complexes, catalysis and UV/Vis and NMR spectroscopy. One of his hobbies was the permanganate taken over from his PhD teacher.^[83]^ *Evert Jan Baerends* (right, *1945), born in a Dutch village, studied chemistry at the Vrije Universiteit Amsterdam, where he entered a career in academia that led him to the chair of theoretical chemistry (1981–2010). Thereafter he worked for several years at Pohang University in South Korea. He developed density functional and density matrix functional theories and led the coding for application of density functional theory to molecules and periodic systems in one, two and three dimensions, and to chemical bonding analysis, initiated by Tom Ziegler. The research tools ADF and BAND are useful in spectroscopy, transition metal and heavy elements chemistry, and in the study of bulk crystals, polymers, and surfaces.^[75]^

When this model was applied to interpret and complement the deductions drawn from experimental studies of complexes with *transition metal atoms in high oxidation states*, such as permanganate or permanganyl fluoride, inconsistencies appeared. In 1958, Ballhausen and Liehr^[72]^ therefore performed various types of approximate and empirically adjusted quantum‐chemical calculations which all resulted in a different order of the molecular orbital energies. A decade later Ballhausen^[71]^ stressed that it is “very likely that configuration interaction between the excited states is important”, and deplored: “As to the placement [of spectral lines] on the energy scale we cannot expect any help from the available calculations”. After half a century of quantum chemical endeavors,[^20^, ^36^, ^37^, ^38^, ^39^, ^52^, ^72^, ^73^, ^74^] Ziegler^[58]^ (Figure 12, left) still noted that: “It would be very interesting to have a study based on CASPT2”, a more reliable technique which requires very extensive calculation efforts. Substantiating Ballhausen's early discontent, Buijse and Baerends provided indications that any one‐electron orbital approach, be it ab‐initio Hartree–Fock, density‐functional Kohn–Sham or semi‐empirically corrected NDO self‐consistent field approaches, will cause artifacts.^[75]^

**Scheme 3:**
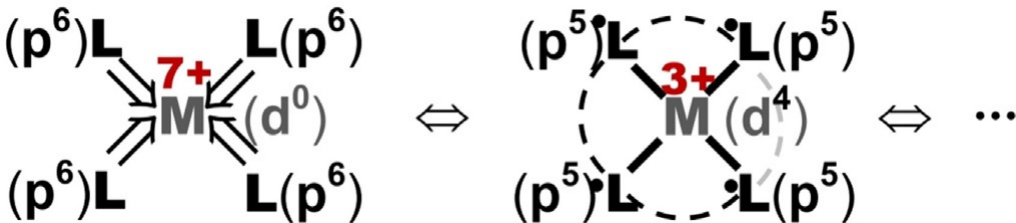
Left: A closed‐shell complex of a late 3d transition‐metal atom M with formal d^0^ electron configuration and four ligands L, σ and π dative‐bonding by their formal p^6^ closed‐shells. Middle: Due to the high effective electronegativity of the formally naked M(d^0^) cation, electronic charge is attracted from the ligands toward M. The M‐d shell becomes effectively partially occupied (for example d^4^) and the four ligands carry partial holes (for example p^5^), which enables covalent M−L and L−L bond formation in the case of a small cationic radius, leading to ligand–ligand overlap. The formal charges given in red (+7 and +3) refer to the case for M = Mn with its corresponding 3d population (d^0^ and d^4^). Right: Many similar “resonating” structures are possible, giving rise to an extremely complex configuration mixing with a break‐down of the common localized orbital picture and the corresponding set of Lewis structures.

The results of Buijse and Baerends suggest that any compound of the *later 3d transition metals in a high oxidation state will exhibit this specific electronic structure*. Common formalisms feature closed shell cores 1s^2^2s^2^2p^6^3s^2^3p^6^ *only apparently* without (or with very low) d electron population, and closed shell ligands (halogenide or chalcogenide anions). The (partially occupied) 3d valence shell is largely located inside the M core–shell that becomes significantly “polarized” statically and dynamically upon M(d)−L(p) bond formation. The anionic ligands are also easily deformed, both meaning significant dispersion interactions that require the simulation of extended *dynamic* many‐electron correlations. The repulsions between the closed metal and ligand core–shells lead to stretched M(d)−L(p) electron‐pair bonds, causing strong *static* electron correlation. In addition, the high oxidation state of the metal increases its effective electronegativity, inducing ligand to metal charge transfer in the molecular ground state and some electron‐hole character in the ligand shells. The latter enables *ligand–ligand bonding* ^[76]^ in cases of late 3d transition metal atoms in high oxidation states with small effective radii,[^77^, ^78^] which requires the simulation of a huge amount of static many‐electron correlations (Scheme 3). This ligand–ligand bonding phenomenon applies for example, also to the case of the hypothetical FeO_4_ molecule and the [FeO_4_]^2−^ dianion, where oxide ligands under the influence of the Fe(VIII/VI) centers may be combined to peroxide ligands leading to ready decomposition with loss of dioxygen.[^1^, ^76^]

Buijse and Baerends^[75]^ noted that in these cases the simplistic individual‐electron or ‐orbital picture causes some molecular orbitals to become polarized too much toward the central atom, and other orbitals toward the ligands, so that the charge distribution becomes artifactually biased and the bonding appears too ionic. State‐of‐the art medium‐sized ab‐initio CAS‐SCF^[36]^ or single‐reference CCSD(T)^[79]^ approaches yield insufficiently accurate structures and stabilities. Often the computationally much cheaper density functional approaches such as BP86, B3LYP, M06L, CAM‐B3LYP etc., give satisfactory structures, energies and vibrational frequencies (in particular if semi‐empirical correction factors for the involved bond types are introduced). The correct description of electronic excitations yet remains a theoretical challenge as mentioned half a century ago.

For interpretative purposes of experimental data, orbital models at various levels of the density functional approximation may still be helpful, but require a careful empirically based selection. A typical alternative orbital level diagram is shown in Scheme 2 on the right hand side. There are now five low‐energy molecular orbitals, the main difference being their nearly homopolar covalent M‐3d+L‐2p(σ,π) character. The e and t_2_ levels are energetically close, while the a_1_ level is higher and only weakly bonding due to the small admixture of the rather diffuse M‐4s. The upper part of the occupied molecular valence band contains six non‐bonding ligand‐dominated orbitals, with t_1_ either above or below t_2_, like on the left hand side. The second main difference is the inverted order t2* < e* < a_1_* of the antibonding orbitals, and their strongly mixed M(d)‐L(p) character.

This description of permanganate as a case with an “inverted ligand field” can be found already in the early work of Jasinski, Holt, Hood, and Moskowitz of 1975 based on SCF‐Xα‐SW calculations. These authors also considered permanganyl fluoride under the symmetry‐reduction from *T_d_* to *C*_3*v*_, and based on the revised diagram the UV/Vis absorption characteristics have tentatively been assigned for both MnO_4_^−^ and MnO_3_F.^[32]^ Similar results were obtained in 1994 by the group of P. Decleva using ab initio CI methods.^[39]^

Over the past 70 years, more or less crude density‐functional or semi‐empirical or ab‐initio orbital‐calculations on Cr^VI^, Mn^VII^ or Fe^VIII^ complexes yielded orbital energy patterns as on the left or right side of Scheme 2, i.e. (i) a minor admixture of M‐3d to the lower e,t_2_ part of the ligand‐dominated valence band, (ii) a corresponding minor admixture of ligand character to the spectroscopic e*,t_2_* upper valence band, (iii) the a_1_M‐4s+L‐2pσ orbital more or less bonding, that is, nearer to the lower or upper part of the valence band, (iv) with a similar variation of the antibonding a_1_* partner in the spectroscopic upper range, and (v) most importantly either the normal e* < t_2_* order,[^32^, ^72^, ^80^] the inverted ligand field order t_2_* < e*,[^39^, ^74^, ^75^, ^81^, ^82^, ^83^] or the near‐degenerate situation e* ≈ t_2_*.^[75]^ In more recent years, extended ab‐initio approaches and computer network power have become efficient enough to produce more reliable data in the future. The huge computational effort for such small molecules however may only be acceptable for really important questions that cannot yet be solved experimentally. Concerning the title compound MnO_3_F, its qualitative molecular orbital level scheme may then probably be derived from the right side of Scheme 2 with adaption to the appropriate symmetry (*T_d_* → *C*_3*v*_).^[32]^ In summary, the “weird molecule” MnO_3_F has been well taken care of experimentally, while it remains a true challenge for theoretical approaches as to its electronic structure.

## A Personal Note

More than 60 years ago, one of the authors (HS) prepared trimethylsilyl *perrhenate*, Me_3_SiOReO_3,_ the first ester of perrhenic acid, as a highlight of his doctorate thesis.^[84]^ The compound was found to be a stable crystalline material, and its crystal structure has later been determined by the “Sheldrick brothers”.^[85]^ However, all subsequent attempts to prepare trimethylsilyl *permanganate* Me_3_SiOMnO_3_ failed and ended up in fierce explosions depositing for quite some time pieces of shattered glass in the face and extremities of the experimentalist owing to incomplete safety precautions. When similar accidents happened with bis(trimethylsilyl) chromate, (Me_3_SiO)_2_CrO_2_, this exercise was brought to a hold by the supervisor. The experience left the student for the rest of his career with an admiration for those brave scientists who carried on to contribute to this challenging chemistry (above).

## Conflict of interest

The authors declare no conflict of interest.

## Biographical Information

*W. H. Eugen Schwarz received a diploma in experimental physical chemistry from Hamburg University and a PhD in natural philosophy from Frankfurt University. From 1976 to 2002 he was professor of theoretical chemistry at Siegen University. He worked on quantum chemical theory and applications (atomic core potentials, relativistic effects etc.), on the elucidation of basic chemical concepts (bonding, periodic table) and on the history and philosophy of chemistry. As an emeritus professor he is still active at the theoretical chemistry center of Tsinghua University Beijing*.

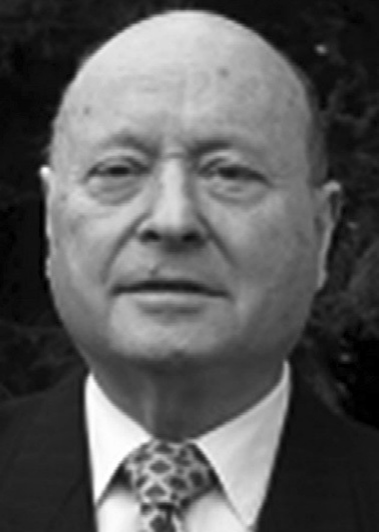

## Biographical Information

*Hubert Schmidbaur received his diploma and PhD in chemistry at the University of Munich (LMU) and started his academic career at the universities of Marburg and Würzburg before he joined the Technical University of Munich (TUM) in 1973, where he served to his retirement in 2003. As an emeritus he since had numerous appointments abroad. Following work in diverse fields of inorganic, organometallic and bioinorganic chemistry he has dedicated much of his research to the chemistry of gold*.